# Effect of Interdental Enamel Reduction on Clinical Attachment Loss, Bleeding on Probing, and Incidence of Caries in Treating Class I Malocclusion Cases: A Retrospective Cohort Study

**DOI:** 10.7759/cureus.35018

**Published:** 2023-02-15

**Authors:** Majid Shalchi, Naghmeh Abdollahi, Elaheh Shafiei Haghshenas, Samar Khabbaz, Pooya Olyaee

**Affiliations:** 1 Department of Orthodontics, Guilan University of Medical Sciences, School of Dentistry, Rasht, IRN; 2 Dental Research Laboratory, Howard University College of Dentistry, Washington, USA; 3 Department of Orthodontics, Kashan University of Medical Sciences, School of Dentistry, Kashan, IRN; 4 Department of Orthodontics, Urmia University of Medical Science, School of Dentistry, Urmia, IRN

**Keywords:** clinical attachment loss, bleeding on probing, caries, fluoride, stripping, interdental enamel reduction

## Abstract

Introduction

Interdental Enamel Reduction (IER) is a clinical procedure that reduces the mesiodistal size of permanent teeth by enamel removal and anatomical re-contouring. The aim of this study was to investigate the effect of IER on patients’ gingival health status, including clinical attachment loss (CAL) and bleeding on probing (BOP). Furthermore, in this study, the incidence of caries after IER with or without fluoride therapy was evaluated.

Methods

In this retrospective cohort study, 90 patients who had started and completed their orthodontic treatment within the past two years were divided into three groups as follows: In group 1, patients had received interproximal stripping on their anterior mandibular teeth. Patients in group 2 had also received interproximal stripping on their mandibular anterior teeth and topical fluoride had been applied after IER. Patients in group 3 had only received orthodontic treatment without any interproximal stripping. Then, patients were examined for CAL, BOP, and incidence of caries.

Results

CAL for patients in the IER and control groups were 2.06±0.18 and 2.08±0.16, respectively. Also, BOP for patients in the IER and control groups were 3.01±0.14 and 3.05±0.19, respectively. Incidences of caries, BOP, and CAL were not significantly different between the group of patients who received IER and the control group (P>0.05). Moreover, the incidence of caries was not significantly different between the patients who received topical fluoride after IER and those who did not receive fluoride (P=0.999).

Conclusion

Interproximal stripping of mandibular anterior teeth before orthodontic treatment does not significantly increase the incidence of caries, BOP, and CAL. Moreover, the application of topical fluoride after IER has no significant effect on the incidence of caries.

## Introduction

Interdental enamel reduction (IER) or interproximal stripping is a clinical procedure that reduces the mesiodistal size of permanent teeth by enamel removal and anatomical re-contouring [1]. In recent years, many orthodontists have increasingly focused on non-extraction treatments, and IER is being widely used by clinicians and researchers to obtain space in orthodontic treatment [2]. IER is performed using different methods including abrasive strips, diamond-coated disks, tungsten carbide, and diamond burs [3].

Since IER is irreversible, a careful and precise evaluation before the procedure is necessary [4]. IER creates a large amount of groove on the interdental surfaces of the teeth, which will remain after conventional polishing methods. These grooves cannot be cleaned by flossing and brushing and are susceptible to bacterial adhesion and biofilm accumulation [5].

Severe IER may remove a significant thickness of the enamel and lead to increased dental sensitivity, pulpal irreversible damage, plaque formation, and dental caries [6-8]. The other concern is periodontal breakdown. Orthodontic treatments can increase periodontal indices including pocket depth and bleeding on probing (BOP) [9]. It has been explained that after IER, the roots might come too close and that leads to reduced interdental alveolar bone septa, which may cause attachment loss and more periodontal problems [3].

Previous microscopic studies have shown that IER causes damage to the enamel surface and increases the susceptibility to demineralization [10,11]. In order to prevent the progression of enamel demineralization, fluoride application has been suggested [12]. It has also shown that fluoride can facilitate tooth remineralization [13].

The aim of this study was to investigate the effect of IER on patients’ gingival health status, including clinical attachment loss (CAL) and BOP. Furthermore, the incidence of caries after IER with or without fluoride therapy was evaluated.

## Materials and methods

This retrospective cohort study was approved by the Ethics Committee of the Dental Research Center at Guilan University of Medical Sciences, Rasht, Iran. After reviewing the patients' charts in the orthodontic department of Guilan School of Dentistry, 90 qualified patients who started and completed their orthodontic treatment within the past two years and satisfied the inclusion criteria were contacted and recalled to participate in the study. Therefore, the duration of the treatment for all patients was less than two years.

The patients were included in the study if they had Class I malocclusion, and their photographs, radiographs, and clinical records were available to determine the periodontium condition, including their BOP and CAL, and caries at the beginning of the study. 

All photographs and radiographs had been taken in the photography center and radiology department of Guilan University of Medical Science, School of Dentistry.

Patients with the following criteria were excluded from the study: taking any medicine affecting the oral cavity within the last two years, history of smoking, having a periodontal pocket depth of more than 3 mm, having CAL before the treatment, or having active dental caries at the beginning of their orthodontic treatment.

Ninety patients satisfying the inclusion criteria were recalled to the School of Dentistry and written consent was obtained. Patients were divided into three groups of 30. (1) Group 1 included patients who had received interproximal stripping on their six anterior mandibular teeth (mesial and distal) before their orthodontic treatment with the same instrument (6 mm wide, medium double-sided IPR Diamond strips, VOKODAK Medical Equipment, China); 0.1 mm IER was done on each surface. (2) Group 2: Patients in this group had also received interproximal stripping on their mandibular anterior teeth, but in this group, topical fluoride, APF thixotropic gel (acidulated phosphate fluoride, 1.23% fluoride ion, Gelato, Keystone, USA) was applied immediately after IER. In this group, following the IER, the teeth had been rinsed and dried thoroughly, and the fluoride gel had been applied and remained on the teeth for one minute. Then, the gel was rinsed and the patients were requested to avoid eating and drinking for at least one hour. The application of the gel was not repeated. (3) Group 3: In this group, patients had only received orthodontic treatment without any interproximal stripping (control group). All patients in the three study groups were treated with a fixed orthodontic appliance (slot 0.022 × 0.030”, 3M Unitek, Monrovia, California), and no removable orthodontic appliance or aligner was utilized. All the procedures were performed by the postdoctoral orthodontic students, under the supervision of a certified orthodontist.

In order to evaluate periodontal condition, CAL and BOP were assessed again after recalling the patients using William’s probe (CP-12/thin Williams color-coded probe, Hu-Friedy, Chicago, IL). CAL was measured at 6 points (mesiobuccal, buccal, distobuccal, mesiolingual, lingual, and distolingual) around the six mandibular anterior teeth, as the distance from the cementoenamel junction (CEJ) to the base of the pocket and their mean was reported in mm [14]. To assess BOP, the periodontal probe was moved through the gingival sulcus of the mandibular anterior teeth, the presence of bleeding after 30 seconds was considered positive BOP. Therefore, in terms of BOP, each patient was scored from 0 to 6 (0 means no tooth showed BOP, and 6 indicates all six mandibular anterior teeth had BOP) [14]. All measurements were performed by one postdoctoral orthodontic student and one certified orthodontist, and if their findings were different, the mean of those numbers was recorded. 

Dental caries in the mesial and distal surfaces of six mandibular anterior teeth (1080 surfaces) were evaluated using parallel periapical radiographs (PSP sensor, Digor Optime, Finland) and was recorded as score 0 (no caries), score 1 (caries in the enamel), and score 2 (caries in the dentin) [15].

Statistical analysis

Data were analyzed using SPSS version 24 (IBM Corp, Armonk, NY). The analysis of variance (ANOVA) test and the Tukey test were used for quantitative variables, and the chi-square test and Fisher's exact test were used for qualitative variables at the significance level of 0.05.

## Results

The mean age of patients in the three groups was 24.7±6.09, 26.33±4.86, and 20.93±5.41 years old, respectively. All patients had class I malocclusion. The baseline characteristics of participants are presented in Table 1.

**Table 1:** Baseline sample characteristic

Study groups	Mean age (P>0.05)		Bleeding on probing		Clinical attachment loss before orthodontic treatment	Active caries before orthodontic treatment	Gender			
							Female	Male	P-Value for each study group	
	Year-old	P-Value	Score before orthodontic treatment	P-Value						
First group (IER)	24.7±6.09	P>0.05	1.82±0.98	P>0.05	0	0	46.7% (14)	53.3% (16)	P>0.05	
Second group (Strip+ Fluoride)	26.33±4.86		2.01±0.81		0	0	60% (18)	40% (12)	P>0.05	
Third group (Control)	20.93±5.41		1.92±0.51		0	0	60% (18)	40% (12)	P>0.05	

BOP and CAL were assessed in the present study. Based on the results, BOP and CAL were not significantly different among the patients in the three study groups (P>0.05). Table 2 demonstrates the mean CAL and BOP scores of the patients in the three study groups.

**Table 2:** The mean clinical attachment loss and bleeding on probing score of six mandibular anterior teeth, after completion of orthodontic treatment, for three study groups

Study groups	Clinical attachment loss		Bleeding on probing	
	Millimeter	P-Value	Score	P-Value
First group (IER)	2.06±0.18	(P>0.05)	3.01±0.14	(P>0.05)
Second group (IER+ Fluoride)	2.02±0.09		3.03±0.07	
Third group (control)	2.08±0.16		3.05±0.19	

Dental caries was also assessed and the findings indicated that there was no significant difference among the three study groups in terms of caries (P=0.999) (Table 3).

**Table 3:** The incidence of caries, after completion of the orthodontic treatment, in three study groups

Study groups	P-Value	Caries		
		Score 0 (No caries)	Score 1 (Caries in enamel)	Score 2 (Caries in dentin)
First group (IER)	(P>0.05)	99.7% (1077)	0.3% (3)	0% (0)
Second group (IER+ Fluoride)		100% (1080)	0% (0)	0% (0)
Third group (control)		100% (1080)	0% (0)	0% (0)

## Discussion

The present study demonstrates that IER during orthodontic treatment does not cause the teeth to become more susceptible to caries and periodontal disease.

The stripping technique might slightly damage the enamel and create scratches and furrows on the tooth surface [16], leading to microbial plaque accumulation [7]. However, it seems that patients’ saliva induces remineralization and restores the affected surfaces [17]. That is why, in this study, IER did not increase the risk of caries on the treated teeth. Similarly, Kortesi et al. performed a systematic review and concluded that the incidence of caries on teeth that were previously treated by IER was the same as that on intact teeth [18].

This study also showed that IER did not significantly affect the periodontal condition (BOP and CAL) of the treated teeth. It is known that one of the concerns regarding IER is its effect on plaque accumulation and periodontal disease [4]. It has also been explained that IER might decrease the horizontal distance between the roots leading to reduced interdental alveolar bone septa and CAL [3]. However, our findings indicated that IER did not increase periodontal breakdown. Zachrisson et al. stated that interdental gingival retraction is reduced after IER and intact gingival papillae form between the mandibular incisors following the resolution of anterior crowding [19]. Also, the other studies that evaluated the alignment of crowded anterior teeth with different techniques did not report any significant change in the periodontal indices after orthodontic treatment [20,21]. Therefore, arranging the teeth and moving them into their ideal position after IER and orthodontic treatment might decrease plaque accumulation and that explains why BOP was not increased in those patients. Furthermore, a previous study has shown that the horizontal distance between the mandibular incisor roots did not decrease after IER [3]. The authors explained that the incisor roots in untreated patients with anterior crowding are probably closer together, and orthodontic treatment following IER can arrange the teeth and improve their position and even increase their roots’ horizontal distance [3]. Therefore, there is no significant logic indicating IER leads to CAL and an increase in BOP. This is also in accordance with previous studies [1-4,6,22].

The efficiency of the application of fluoride after IER is a controversial issue. Zachirsson et al. considered the use of fluoride after IER to be unnecessary [23]. Lapenaite et al. noted that the use of topical fluoride gel in patients who use fluoride toothpaste and fluoride-containing water on stripped surfaces has no benefit [4]. On the other hand, there are multiple studies suggesting the use of fluoride gel after IER to protect enamel [8,11,24,25]. 

Our findings indicated that the application of fluoride after IER did not lead to any significant change in patients’ caries incidence. As mentioned above, patients’ saliva allegedly induces adequate remineralization to protect enamel after IER [17]. Therefore, the use of a high-concentration fluoride gel after enamel may not significantly affect this remineralization process.

Limitations

One of the limitations of the present study is its sample size. This study was a retrospective study and was performed on the completed orthodontic cases that received their treatments in the orthodontic department of the Guilan School of Dentistry within the past two years. Therefore, the available cases that met the inclusion criteria were limited.

Furthermore, in the current study, the duration of the treatment for all patients was less than two years but was not exactly the same for all participants. Therefore, this factor might affect the periodontal condition of the patients. Also, having a longer duration of treatment means those patients finished their treatment recently and might have a higher BOP score compared to the patients who finished their treatment, for example, three months ago.

## Conclusions

Based on the results of this study, interproximal stripping of mandibular anterior teeth before orthodontic treatment does not significantly increase the incidence of caries, BOP, and CAL. Moreover, the application of topical fluoride after IER has no significant effect on the incidence of caries.

More research is needed to assess the other potential side effects of IER, including postoperation sensitivity and plaque accumulation. Also, it is recommended to evaluate the effect of IER on the other teeth in patients' dentition.
